# Advanced-Glycation End-Products Induce Podocyte Injury and Contribute to Proteinuria

**DOI:** 10.3389/fmed.2021.685447

**Published:** 2021-07-01

**Authors:** Rajkishor Nishad, Vazeeha Tahaseen, Rajesh Kavvuri, Manga Motrapu, Ashish K Singh, Kiranmayi Peddi, Anil K Pasupulati

**Affiliations:** ^1^Department of Biochemistry, University of Hyderabad, Hyderabad, India; ^2^Department of Biochemistry, Acharya Nagarjuna University, Guntur, India

**Keywords:** diabetic kidney disease, advanced glycation end-products, carboxy methyl lysine, podocytes, albuminuria, Introduction, diabetic nephropathy

## Abstract

The prevalence of diabetes reaches epidemic proportions. Diabetes is the leading cause of end-stage kidney disease (ESKD) since 30–40% of diabetic patients develop diabetic nephropathy. Albuminuria and glomerular filtration rate used to assess kidney function are considered surrogate outcomes of chronic kidney disease. The search for a biomarker that predicts progression to diabetic kidney disease is intense. We analyzed the association of serum advanced glycation end-products (AGEs) index (AGI) with impaired kidney function in poorly controlled type II diabetic patients. We observed an association between AGI and impaired kidney function in microalbuminuria patients with hyperglycemia. A significant association between AGEs, particularly carboxymethyl lysine (CML), and impaired kidney function were observed. Administration of AGEs to mice showed heavy proteinuria and glomerular abnormalities. Reduced podocyte number in mice administered with AGEs could be attributed to the epithelial-mesenchymal transition of podocytes. Our study suggests CML could be independently related to the podocyte injury and the risk of DN progression to ESKD in patients with microalbuminuria. AGEs in general or CML could be considered a prognostic marker to assess diabetic kidney disease.

## Highlights

- Advanced glycation end-products (AGEs) index is associated with proteinuria among diabetic subjects.
- Carboxymethyl lysine, a well-characterized AGE, correlated with podocyte injury.
- Administration of AGEs to mice manifested in EMT of podocytes and impaired kidney function.
- Inhibitor against receptor for AGEs prevented podocyte injury and improved proteinuria.

## Introduction

Diabetes has long been a growing epidemic, and Asia accounts for 60% of the world's diabetic population (1, 2). The increased prevalence of diabetes led to a surge in macro and microvascular complications such as visual impairment, coronary heart disease, stroke, neuropathy, and diabetic nephropathy (DN). DN is a chronic disease that accounts for 44% of new end-stage kidney disease (ESKD) cases, with 6% attributed to type I and 38% attributed to type II diabetes (3). It was projected that an increase of 3 million DN cases over 20 years (3). DN clinical manifestations include glomerular transient hyperfiltration, proteinuria, kidney hypertrophy, fibrosis, and decreased glomerular filtration rate (GFR) (4). During the early DN stage, a patient shows hyperfiltration, represented by a rise in GFR and occasional microalbuminuria (ratio of urine albumin to creatinine ≥ 30 mg/g) (4). The DN's progressive stage is represented by a gradual decline of the GFR, persistence of microalbuminuria, and subsequent macroalbuminuria (ratio of urine albumin to creatinine ≥ 300 mg/g). The advanced DN stage is characterized by severe proteinuria and chronic kidney insufficiency that ultimately manifest in ESKD. Both albuminuria and impaired GFR are the strongest predictors of progression to ESKD in patients with diabetes.

Biomarkers play an essential role in the early detection of DN and its progression to ESKD, whereas microalbuminuria is one of the predominant markers (5). Microalbuminuria also indicates generalized endothelial dysfunction and suggests kidney involvement with cardiovascular and cerebral impairment (6). Microalbuminuria is considered an early stage of, rather than a predictor of, DN and subsequent kidney impairment. Furthermore, microalbuminuria reflects not only glomerular injury but also tubular lesions (5). Although microalbuminuria is accepted to indicate potential renal damage, it is neither an accurately predict progression of diabetic kidney disease nor reliably be used to track response to therapy (7). Regression from microalbuminuria to normoalbuminuria is more likely than progression toward overt proteinuria (8). There is a growing need for a more dependable and reliable biomarker for both predicting and tracking DN.

Among the myriad hemodynamic, metabolic, and inflammatory factors that participate in DN's pathogenesis, persistent hyperglycemia is predominant. It is noteworthy that a strong relationship between poor glycemic control and DN exists (9, 10). Prolonged hyperglycemia ensures the formation of advanced glycation end-products (AGEs) in the kidney and other sites of diabetic complications (11). AGEs comprise heterogeneous compounds formed during a series of non-enzymatic (Maillard) glycation (NEG) reactions between the amino group of proteins, lipids, and nucleotides with reducing sugars (12–14). DN patients with macroalbuminuria and patients on hemodialysis had significantly higher serum AGEs than those with microalbuminuria (15). One of the most widely studied AGEs is carboxymethyl-lysine (CML) and is being used as markers for *in vivo* formation of AGEs (14, 16, 17). CML has been used as a biomarker for long-term protein damage. Elevated tissue CML concentrations are associated with kidney and retinal complications in patients with diabetes (18, 19).

In the case of DN, early screening and evaluation of the kidney injury may help assess the risk of ESKD and strategizing the therapeutic regimen. Although glycated hemoglobin (HbA1c) has proven to be a reliable prognostic marker in the general diabetic population, it may not be valid in patients with diabetes and chronic kidney disease (20). It is debated whether HbA1c corresponds to the same mean glucose concentrations in people with ESKD (21, 22). Further, HbA1c is influenced by several factors, including the RBCs' lifespan, administration of erythropoietin, uremic environment, and blood transfusions (20, 23, 24). In contrast, glycated albumin (25) is suggested as a preferred marker for assessing glycemic control in advanced chronic kidney disease only (20). According to UK prospective diabetes study (26), intensive blood-glucose control in patients with type II diabetes reduces microvascular complications, particularly in patients with a diabetic kidney disease whose cardiovascular risk increases with worsening proteinuria (23, 26). Therefore, a biomarker that could predict impaired kidney function in patients with poor glycemic control and microalbuminuria would help manage DN effectively. Accumulation of serum AGEs in DN is due to increased accumulation and decreased elimination by the kidney (15). We examined serum and glomerular AGEs association with glomerular injury and macroalbuminuria in patients with DN. Our study identified glomerular CML levels correlate significantly with epithelial-mesenchymal transition (EMT) of glomerular podocytes and glomerulosclerosis in patients with DN.

## Materials and Methods

### Materials

The primary antibodies are as follows: anti-E-cadherin (#3195), anti-N-cadherin (#13116), anti-col IV (#PAA180Hu01), and anti-fibronectin (#PAA037Hu01) were purchased from Cloud-clone (Houston, TX). Anti-nephrin (#NBP1-77303) and anti-podocin (#JB51-33) were purchased from Novus Biologicals (Minneapolis, Minnesota). Anti-α-SMA (#ab5694) was purchased from Abcam (Cambridge, MA). We obtained glutaraldehyde solution (#G5882) and other chemicals from Sigma-Aldrich (Bangalore, India).

### Study Population

Study subjects were enrolled from outpatients attending diabetes specialties centers in Vijayawada and Guntur in the state of Andhra Pradesh, India. We recruited 130 subjects with albuminuria ranging from 150 to 450 mg/day. Inclusion criteria are diabetes with more than 5 years, persistently inadequate glycemic control, and proteinuria above 150 mg/day. These subjects are devoid of other diabetic complications such as diabetic retinopathy, diabetic neuropathy, and cardiovascular complications at recruitment. Exclusion criteria were hematuria, clinical and laboratory findings suggestive of non-diabetic glomerulopathy, and secondary kidney damage due to hypertension. Informed consent was obtained from all individual participants included in the study. This study was carried out following “The Code of Ethics of the World Medical Association (Declaration of Helsinki)” and approved by the Institutional Review Board of Guntur General Hospital, Guntur, Andhra Pradesh, India (GMC/IEC/120/2018).

### Clinical Examination

Anthropometric measurements, including weight, height, and waist measurements were recorded for the patients. Body mass index (BMI) was calculated using the formula: weight (kg)/ height (m^2^). Blood pressure (BP) was monitored thrice by a digital oscillometer (Omron Healthcare Co. Ltd. #HEM-7120). Fasting blood glucose (FBG) was estimated in the whole blood using a glucometer (Accu-Chek Aviva, Roche Diagnostics GmbH, Germany). Blood samples (12 h overnight fasting and post-prandial) were collected in heparin tubes and were centrifuged at 3,500 rpm, 4°C for 20 min to separate plasma and RBC. HbA1c was estimated in whole blood using a D-10 analyzer (Bio-Rad#12010405) based on the principle of fully automated boronate affinity assay. We collected 24 h urine and early morning spot urine, and albumin content was determined by kit from BioSystems (Barcelona, Spain).

### AGE Index

The plasma AGE index of the patients was estimated as described earlier by Sampathkumar et al. (27). Plasma samples were diluted to 1:5, 1:10, 1:20, and 1:40 times in 20 mM PBS and AGE-specific fluorescence (Ex:370 nm and Em:440 nm) was recorded using fluorometer (JASCO-FP-4500). The concentration of AGEs is directly proportional to the fluorescence intensity, and the fluorescence intensity at each addition of the plasma sample was curve fitted to a linear regression line. The regression line slope was termed AGE index and expressed as arbitrary units.

### Biopsy Specimens

Archived kidney biopsies of DN patients (*n* = 30) were collected from the pathology lab. Patients who underwent nephrectomy for a localized kidney tumor were selected for the control group (*n* = 30). The non-affected part of the kidney tissue was utilized for histological examinations. Clinical information of these patients was obtained through the medical records available at intensive care units where patients were hospitalized.

### Histopathological Analysis

For histological analysis, the kidney tissue samples were fixed with 4% neutral buffered paraformaldehyde before embedding in paraffin. Paraffin-embedded tissues were sliced at four μm thickness and stained with hematoxylin and eosin (H&E), periodic acid–Schiff (PAS) stain, and Masson's trichrome stain. Kidney specimens were scored by two pathologists blinded each other and to the patients' identification and clinical data. Kidney injury score was assessed according to Tervaert et al. (28). At least six glomeruli were captured for each biopsy sample and quantified for histopathological changes. We took images with a BX51 light microscope (Olympus, Tokyo) with appropriate filters. Histological positive staining intensity was quantified using Image J analysis software (NIH, USA). For transmission electron microscope (TEM) images, kidney cortex tissues were processed as described earlier (25). Images were acquired on a JEM-1400 TEM (Jeol, Peabody, MA) using a Gatanultrascan CCD camera (Gatan Inc, Pleasanton, CA) 2K × 2K resolution and 120kV.

### Preparation of AGEs

Glucose-derived AGEs were prepared as reported earlier (29). Briefly, sterile preparations of BSA (100 mg/mL) were mixed with D-glucose (90 mg/mL) and 1mM sodium azide in 0.4M phosphate buffer, pH 7.6, and incubated for 2 weeks at 37°C. The formation of glucose-derived AGEs was confirmed using non-tryptophan AGE fluorescence (λex:370 nm and λem:400–500 nm) and by Western blotting with AGE-specific antibody.

### Cell Culture

Human podocytes were maintained and differentiated essentially as detailed earlier (25). Differentiated podocytes were treated with AGEs in the presence or absence of inhibitor (FPS-ZM1). Protein lysate and RNA were prepared from these podocytes and used for Western blotting and qRT-PCR. Differentiated podocytes were treated with or without AGEs (100 μg/mL) and AGEs+FPS-ZM1 (1 μg/mL) for 48 h. A wound-healing assay was also performed with podocytes essentially as described earlier (25).

### Animals and Tissues

The Animal experimental procedures were performed in adherence with the Institutional Animal Ethics Committee of the University of Hyderabad. C57BL/6J male mice (6–8 weeks old, 31 ± 3g) were used in this study. These mice were randomly distributed into three groups viz. Control, AGEs, and AGEs+FPS-ZM1 (*n* = 6, each group). FPS-ZM1 is an inhibitor for a receptor for AGEs (RAGE). Mice in the control group received an equal volume of phosphate buffer as a vehicle, whereas the experimental group received i.p. injections of *in vitro* prepared AGEs (10 mg/kg b.w); AGEs, and FPS-ZM1 (1 mg/kg b.w) on a daily basis for 4 weeks. At the end of the experimental period, 24 h urine was collected to measure GFR, albumin, and creatinine levels. Additionally, urine was subjected to SDS-PAGE and stained with silver nitrate to visualize the proteins in urine. Animals were perfused, and kidneys were harvested. Kidney sections from paraffin-embedded tissues were used for immunostaining, and the glomerular lysate was used for immunoblotting and mRNA expression analysis.

### Estimation of Glomerular Filtration Rate (GFR)

GFR was estimated in mice as detailed earlier (25) using a FITGFR Test Kit for Inulin according to the manufacturer's instructions (BioPal, Worcester, MA). Briefly, 5 mg/kg of inulin was injected intraperitoneally, followed by serial saphenous bleeds at 30, 60, and 90 min. Next, serum isolation was done and quantified on a inulin ELISA kit. Serum inulin clearance estimation was performed by the nonlinear regression method using a one-phase exponential decay formula (y = Be-bx), and GFR was calculated {GFR = [(I)/(B/b)]/KW, where I is the amount of inulin delivered by the bolus injection, B is y intercept, b is the decay constant, x is time, and KW is kilo weight of the animal}.

### Statistics

Data are represented as a mean with SD. Statistical analysis between groups was performed by *t*-test using GraphPad prism 6. Relationships between parameters were analyzed using Pearson's correlation coefficient with R version 4.0.3.

## Results

### Advanced Glycation Index Is Associated With Impaired Kidney Function in Type II Diabetic Patients

**Figure 1:**
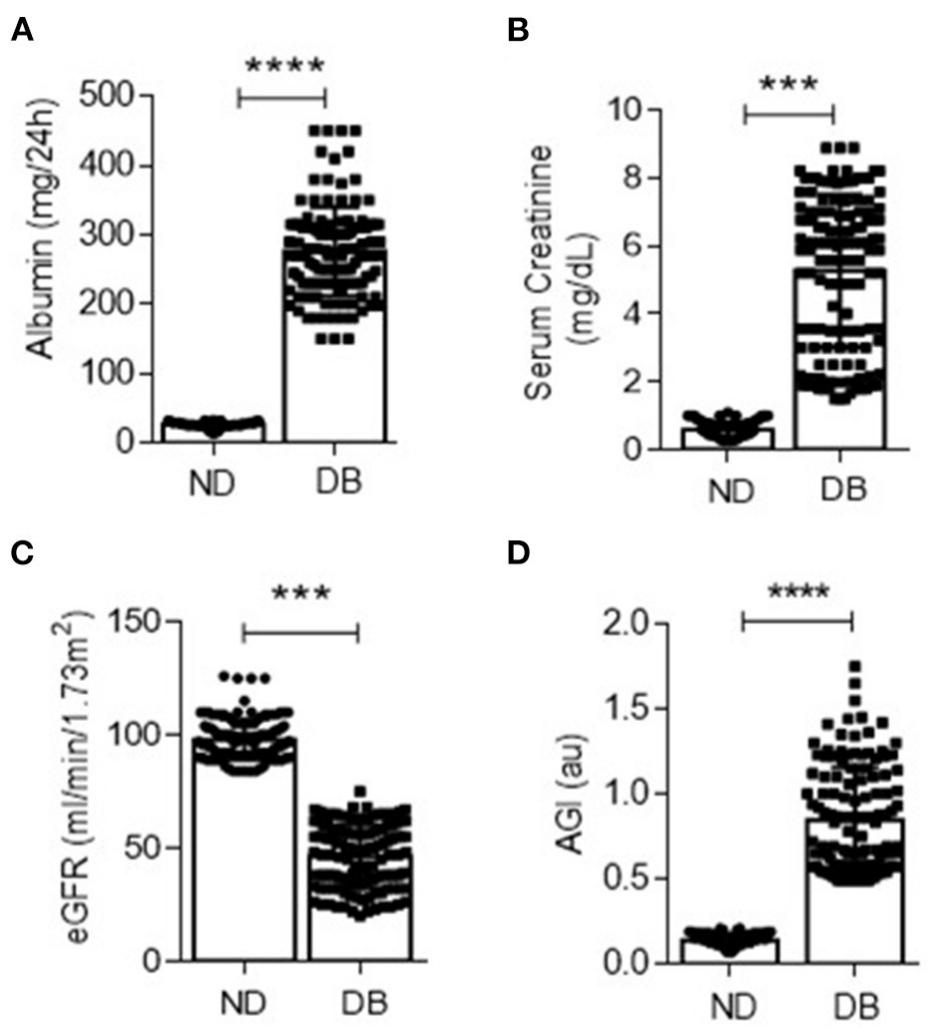
Urinary albumin **(A)** and serum creatinine **(B)** were quantified in non-diabetic (ND) and diabetic (DB) groups. ***p < 0.001 and ****p < 0.0001. **(C)** The estimated glomerular filtration rate (eGFR) was measured in ND and DB groups. ***p < 0.001, *n* = 130 in both the groups. **(D)** Advanced glycation index (AGI) was measured in ND and DB as described in methods. ****p < 0.001, *n* = 130 in each group. Data are the mean ± SD, and statistical significance is calculated by using the student *t*-test.

The clinical characteristics of non-diabetic (controls) and type II diabetic patients are provided in Table 1. The mean age of 130 patients was 56 ± 4.4 years, fasting blood glucose 179 ± 32, post-prandial blood glucose 223 ± 35, BMI 28.2 ± 4.6, and HbA1c 9.75 ± 1.8% (Table 1). The mean urinary albumin (276.87 vs. 25.78 mg/24 h), serum creatinine (5.26 vs. 0.59 mg/dL), eGFR (46.30 vs. 97.86 ml/min/1.73 m^2^), and AGI were significantly different between the controls and diabetic patients (Figures 1A–D). Together, the data suggest that poor glycemic control and AGI in diabetic subjects associate with impaired kidney function.

**Table 1:** Clinical characteristics of the study subjects with and without diabetes.

**Parameter (unit)**	**Non-diabetic**	**Diabetic**
	**(*n* = 130)**	**(*n* = 130)**
Age (yrs)	57 ± 2.3	56 ± 4.4
BMI (Kg/m2)	21.2 ± 3.1	28.2 ± 4.6
Known duration of diabetes (yrs)	N/A	10 ± 3.4
Known duration of proteinuria (yrs)	N/A	2.5 ± 2.8
Fasting glucose (mg/dL)	98 ± 15	179 ± 32
PP Glucose (mg/dL)	110 ± 35	223 ± 35
Systolic blood pressure (mm Hg)	115 ± 18	158 ± 27
Diastolic blood pressure (mm Hg)	79 ± 8	98 ± 6
Serum creatinine (mg/dL)	0.59 ± 0.24	5.26 ± 2.23
Albumin (mg/24h)	25.78 ± 4.25	276.87 ± 64.5
eGFR (ml/min/1.73m2)	97.86 ± 8.98	46.30 ± 13.84
HbA1c (%)	4.5 ± 0.94	9.75 ± 1.8
Glycated albumin (%)	11.45 ± 2.9	28.8 ± 3.2

*Data presented as mean ± SD. p < 0.001. PP, post-prandial; SBP, systolic blood pressure; DBP, diastolic blood pressure; HbA1c, hemoglobin A1c; AGI, glycated albumin index; UACR, urinary albumin to creatinine ratio; eGFR, estimated glomerular filtration rate; au, arbitrary units*.

### Both Serum and Glomerular AGEs Correlate With Decreased Podocin Expression

**Figure 2:**
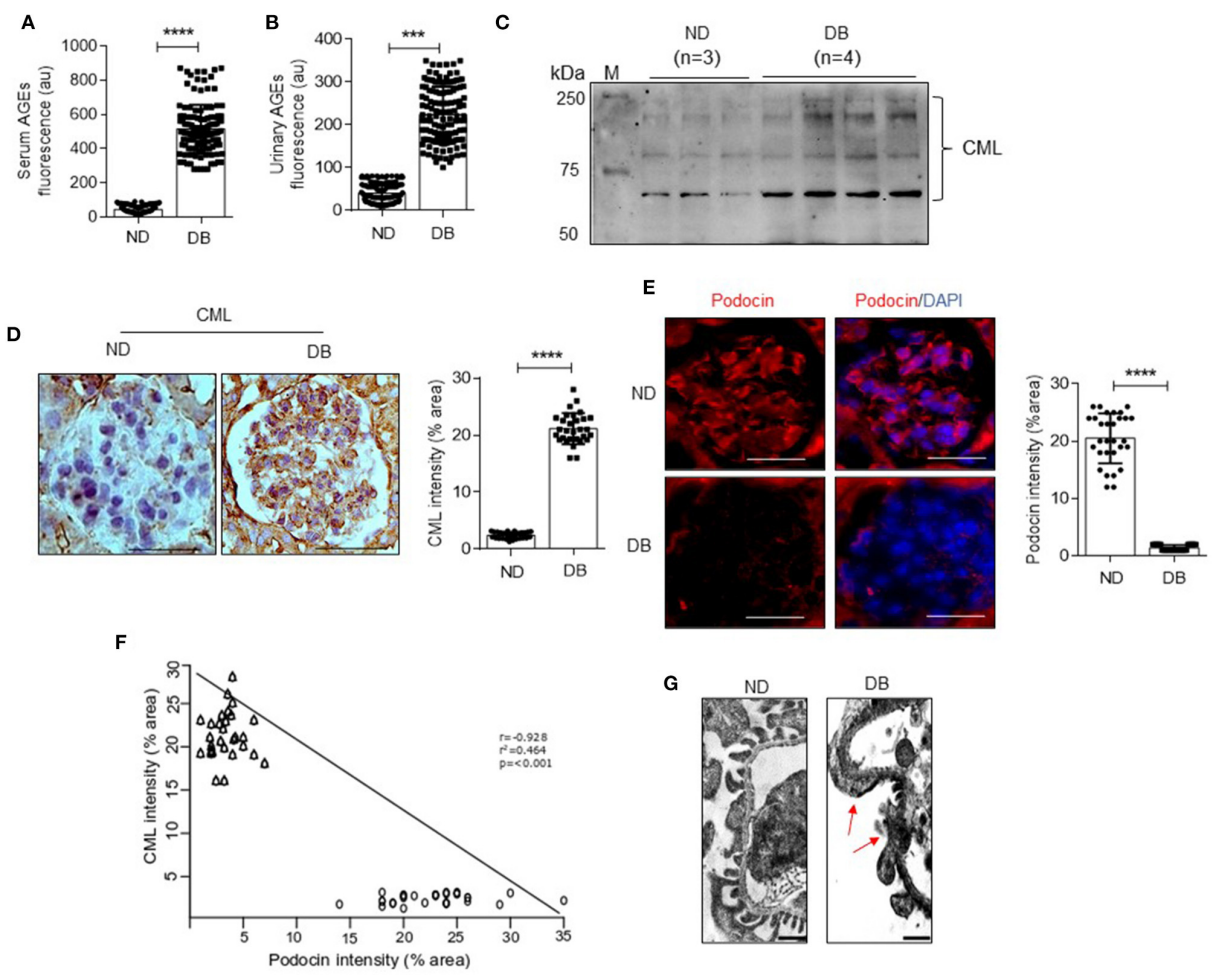
Fluorometric assessment of serum **(A)** and urinary **(B)** AGEs in ND and DB both the groups (*n* = 130) (Ex: 370 nm; Em: 440 nm). ***p < 0.002 and ****p < 0.0001. **(C)** Immunoblotting analysis of CML in serum samples from ND and DB groups. M = standard protein marker. **(D)** Immunohistochemical analysis of CML in the glomerulus of kidney biopsy samples from ND and DB groups (Left). Magnification (x 630). Scale bar = 20 μm. The percentage of stained area per field from glomerulus was quantified and represented as a dot plot (Right). Each data point represents an average value of 6 glomeruli from ND and DB both the groups. ****P < 0.0001. **(E)** Immunohistochemical staining for podocin in the glomerulus of ND and DB kidney biopsy samples (Left). Magnification (x 630). Scale bar = 20 μm. The percentage of stained area per field from glomerulus was quantified and represented as a dot plot (Right panel). Each data point represents the average value of 6 glomeruli from ND and DB both the groups. ****P < 0.0001. The intensity of glomerular expression of CML and podocin was quantified using ImageJ. (**F**) Correlation analysis of CML vs. podocin staining intensity in the glomerulus of ND and DB groups. Correlation is significant at the 0.001 level (2-tailed). (**G**) TEM images of podocytes from ND and DB kidney biopsy samples. The arrow indicates foot process effacement. Scale bars indicate 0.5 μm.

Poor glycemic control in diabetics is presented with excess AGEs (12). Therefore, we assessed the advanced glycation index in serum and urine to determine the status of AGEs empirically, as described earlier (30). AGI was significantly high in diabetic patients and correlated with declined kidney function (Figures 2A,B, 1A,B). Carboxymethyl lysine (CML) is one of the well-characterized AGEs, and elevated CML levels were found in diabetic kidneys and glomeruli [28]. Thus, we determined the extent of AGEs by immunoblotting and immunostaining using an anti-CML antibody. Interestingly, we found elevated CML in both serum (Figure 2C) and glomerulus (Figure 2D) of diabetic patients. Accumulation of CML in diabetic rat glomeruli was proportional with decreased podocyte number (13). Therefore, we stained for podocin, a podocyte-specific marker, and found that decreased podocin expression in glomerular sections from DN patients (Figure 2E). An inverse correlation was observed between CML staining in the glomerulus and decreased podocin expression (Figure 2F). TEM analysis revealed podocyte foot-process effacement (Figure 2G). Together the data suggest that excess AGEs, particularly CML associated with decreased podocin expression, foot-process effacement of podocytes from type II patients with nephropathy.

### Association of Excess Glomerular CML With Epithelial-Mesenchymal Transition (EMT) of Podocytes

**Figure 3:**
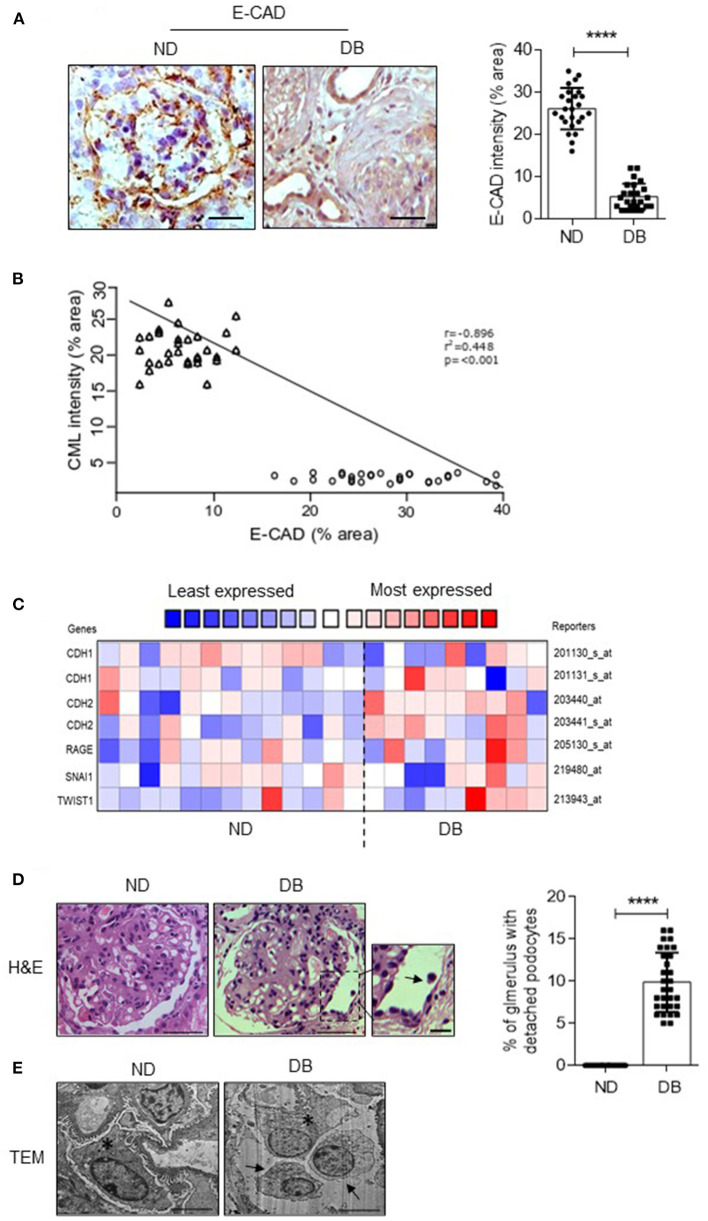
**(A)** Immunohistochemical analysis of E-cadherin in the kidney biopsies sections from ND and DB groups (Left). Magnification (x 630). Scale bar = 20 μm. The stained area (%) per field from glomerulus was quantified, and each data point represents the average value of 6 glomeruli from ND and DB both the groups (Right). ****P < 0.0001. The intensity of glomerular expression of E-cadherin was quantified using ImageJ. **(B)** Correlation between CML vs. E-CAD expression in the glomeruli from ND and DB biopsy samples. Correlation is significant at the 0.001 level (2-tailed). **(C)** Nephroseq analysis comparing *CDH1* (E-cadherin), *CDH2* (N-cadherin)*, RAGE*, and *TWIST* expression levels in non-diabetic (*n* = 13) vs. diabetic individuals (*n* = 9) data set from Woroniecka diabetic glomerulus dataset. **(D)** Hematoxylin and eosin (H&E) staining in glomeruli from ND and DB kidney biopsy samples. A zoomed image representing the detached podocyte (black arrow). Magnification (x 630). Scale bar = 20 μm. Detached podocytes were quantified (%), and each data point represents the average value of 6 glomeruli from *n* = 30 kidney biopsy samples in ND and DB groups. ****P < 0.0001. (Right) **(E)** TEM images of podocyte from ND vs. DB kidney biopsy samples. The asterisks indicate attached podocytes with the glomerular basement membrane, and the black arrow indicates detached podocytes into the urinary space. Scale bars = 2 μm. Data are the mean ± SD, and statistical significance is calculated by using the student *t*-test.

Since excess glomerular CML correlates with reduced podocin number, we next ascertained the mechanisms of podocyte depletion in diabetic patients. An earlier study from our group reported that podocytes undergo EMT in mice administered with CML (2). Therefore, we investigated whether EMT occurs or not in glomeruli from DN patients. E-cadherin (E-CAD, a bonafide marker of epithelial phenotype) expression significantly decreased in DN patients (Figure 3A). A negative correlation was observed between decreased E-CAD expression and the accumulation of glomerular CML staining (Figure 3B). The data derived from Nephroseq (www.Nephroseq.org, University of Michigan, O'Brien Renal Center) also corroborated with our observation that in DN, decreased expression of epithelial marker (E-CAD/CDH1) and increased expression of mesenchymal marker (N-cadherin/CDH2) and transcription factors that elicit EMT phenomenon such as SNAI1 and TWST1 (Figure 3C). Nephroseq data also revealed the upregulation of receptors for AGE (RAGE) in DN patients (Figure 3C). H&E staining and TEM imaging revealed detached podocytes in glomerular space (arrow mark) of DN patients (Figures 3D,E). Together the data suggest podocytes in DN patients undergo EMT, which might be responsible for the observed detached phenotype.

### AGE Index and Decreased Podocyte Count Are Associated With Glomerulosclerosis

**Figure 4:**
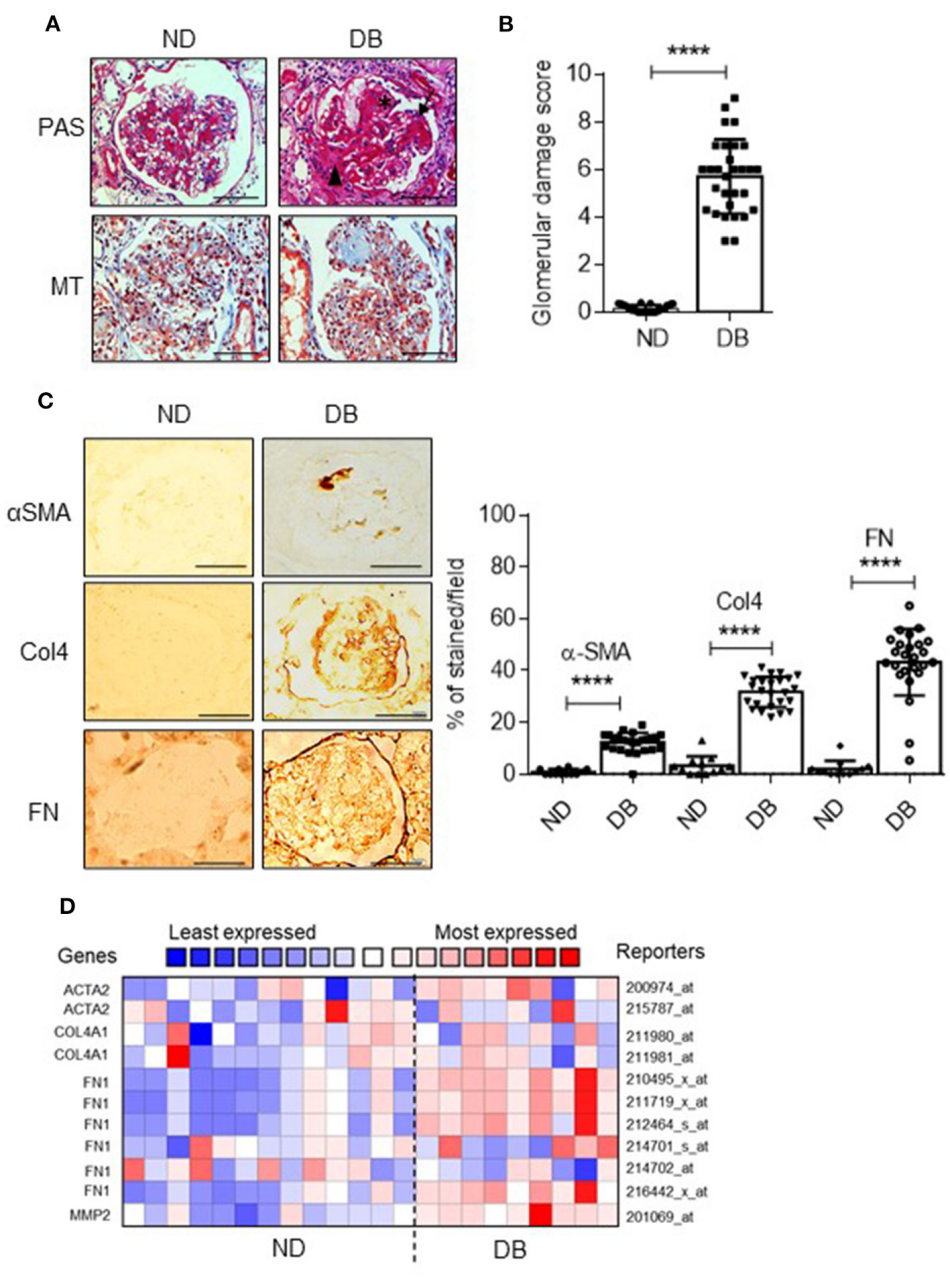
**(A)** Representative images of Periodic acid-Schiff (PAS) and Masson's trichrome (MT) staining in glomeruli from ND and DB patients. Magnification = (x 630). Scale bar = 20 μm. **(B)** Glomerular damage score was derived from PAS-stained images by summing the glomerular capillary blockage (black asterisk), adhesion of glomerular tuft to Bowman's capsule (black arrowhead), and focal segmentation of glomerular tuft (black arrow). Each data point represents the average value of 6 glomeruli from *n* = 30 kidney biopsy samples from ND and DB groups. ****p < 0.0001. **(C)** Representative images of immunohistochemical analysis for Collagen 4, (Col4), α-smooth muscle actin (α-SMA), and fibronectin (FN) in glomerular sections from ND and DB biopsies. Magnification (x 400). Scale bar = 50 μm. The percentage of stained area per field from glomerulus was quantified and represented as a dot plot (right panel). Each data point represents the average value of 6 glomeruli from *n* = 25 kidney biopsy samples in ND and DB groups. ****P < 0.0001. The intensity of glomerular expression of each marker was quantified using ImageJ. **(D)** Nephroseq analysis comparing *ACTA2(*α-SMA), *COL4A1, FN1*, and MMP2 expression in ND (*n* = 13) vs. DB individuals (*n* = 9). Nephroseq data is acquired from Woroniecka diabetic glomerulus dataset.

It demonstrated the correlation of decreased podocyte count with the onset of proteinuria and glomerulosclerosis (2). Since these podocytes counteract the outward forces of glomerular pressure and help maintain the capillary loop's shape, depletion of podocytes leads to bulging of the GBM (31). Additionally, the denuded GBM form a synechia attachment with the parietal epithelial cells and Bowman's capsule, which is thought to ensure focal segmental glomerular sclerosis (FSGS). Since we observed decreased podocyte count in diabetic patients, we assessed the extent of fibrotic changes in the kidney sections. As anticipated, PAS and MT staining revealed significant fibrotic changes in the glomerular region (Figure 4A), concomitant with a high glomerular injury score (Figure 4B). Expression of fibrotic markers such as α-SMA, Col 4, and fibronectin was upregulated in these injured glomeruli as evidenced by immunostaining (Left panel and quantification (Right panel) (Figure 4C). Nephroseq analysis of DN patients also revealed elevated expression of fibrotic markers (Figure 4D). Both our experimental and Nephroseq data suggest that AGE/RAGE activation is associated with glomerular fibrosis.

### Administration of AGEs Manifested in Impaired Kidney Function and EMT of Podocytes Both *in vivo* and *in vitro*

**Figure 5:**
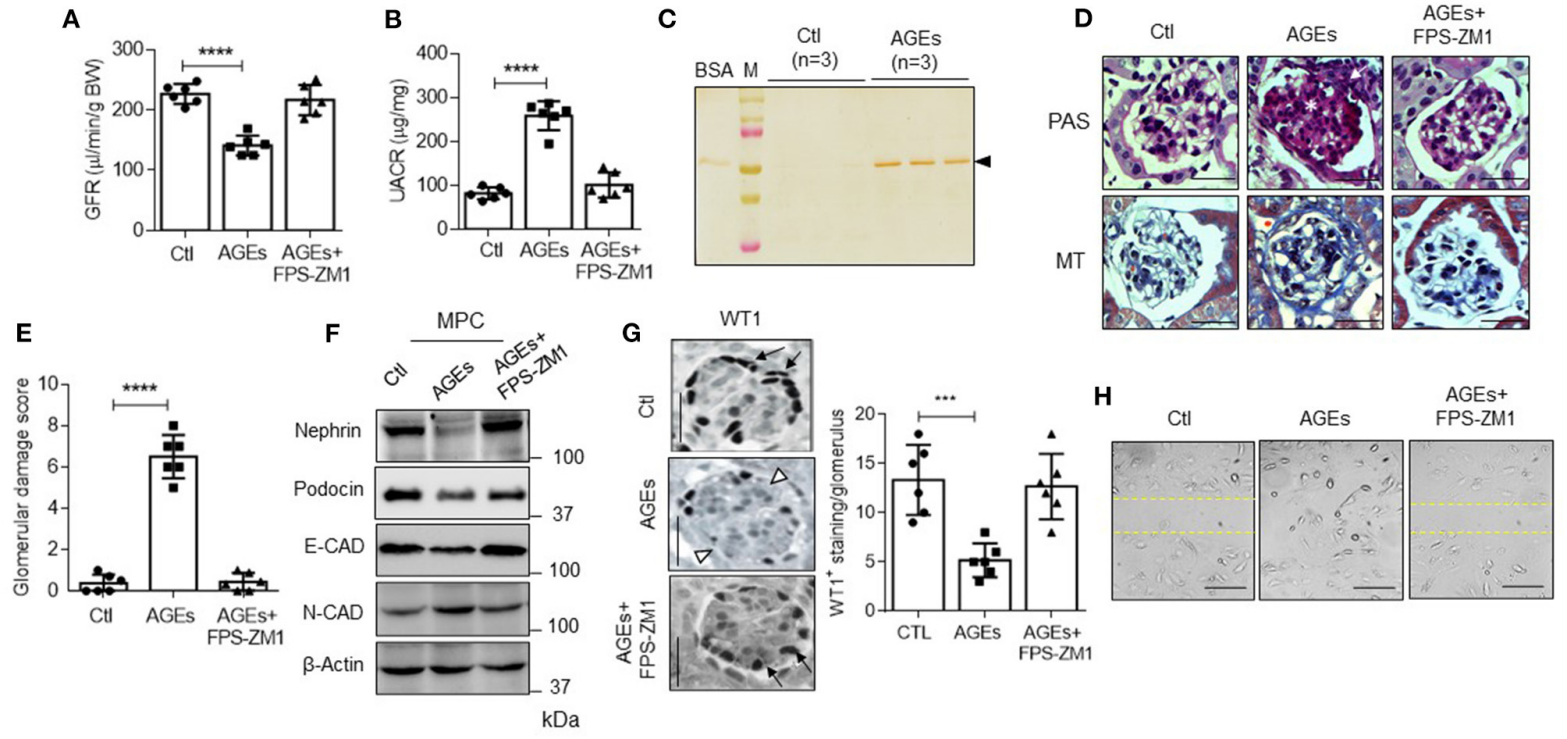
**(A)** GFR and **(B)** UACR in control (Ctl) and AGEs administered mice (*n* = 6). ****p < 0.0001. **(C)** Urinary samples from Ctl and AGEs administered mice were subjected to SDS-PAGE and silver-stained as described under “Experimental Procedures.” BSA was used as a standard. M = standard protein marker. **(D)** PAS and MT staining in glomeruli from with or without AGEs and AGEs+FPS-ZM1 treated mice (*n* = 6). Magnification (x 630). Scale bars indicate 20 μm. **(E)** Glomerular damage score was derived from PAS-stained images by summing the glomerular capillary blockage (white asterisk) and adhesion of glomerular tuft to Bowman's capsule (white arrow). Each data point represents the average value of 20 glomeruli from a single animal from each group (*n* = 6). ****p < 0.0001. **(F)** Immunoblotting analysis for nephrin, podocin, E-CAD, N-CAD, and β-Actin in mice podocytes (MPC) from with or without and AGEs administered mice (*n* = 6). **(G)** Staining for WT1 (podocyte) in glomerulus from mice from each group (Ctl, AGEs, and AGEs+FPS-ZM1). Magnification (x 630). Scale bar indicates 20 μm. The black arrow indicates the podocytes, and the white arrowhead indicates the loss of podocytes in the glomerulus (Left). An average number of WT1 positive (+) cells from 20 glomeruli from each group (Right). ***p < 0.001. **(H)** A wound-healing assay was performed to determine the extent of human podocyte motility from Ctl, AGEs, and AGEs+FPS-ZM1 treatment. Images were captured after 0–12 h treatment using an Olympus inverted microscope. Magnification (x 100). Scale bar indicates 100 μm.

As we observed increased AGI and accumulation of AGEs associated with glomerular injury in patients with type II diabetes, next, we ascertained whether administration of AGEs to mice induces similar pathological features. Administration of AGEs to mice manifested in the GFR decline and albuminuria (Figures 5A–C). PAS and MT staining of paraffin-embedded sections from mice administered with AGEs revealed glomerulosclerosis (Figure 5D). Histological analysis of AGE-treated mice showed that a high glomerular injury (Figure 5E). Further, decreased expression of nephrin, podocin, E-cadherin, and increased expression of N-cadherin were also noticed in mouse podocytes administered with AGEs (Figure 5F). Decreased number of podocytes per glomerulus was observed in these mice administered with AGEs as assessed in WT1 staining (Figure 5G). Human podocytes exposed to AGEs showed enhanced migratory property with decreased epithelial nature (Figure 5H), corroborating our *in vivo* observation that AGEs elicit podocyte EMT and migratory phenotype. Receptor for AGE (RAGE) inhibitor protected the mice from podocyte EMT and glomerulosclerosis (Figures 5D–H). Together, our data suggest AGEs adversely affect kidney function by eliciting podocyte injury and depletion, possibly by promoting podocyte EMT.

## Discussion

The incidence of ESKD is increasing globally, and DN is one of the leading causes. Although eGFR and albuminuria reflect kidney function, these parameters are part of the diagnosis. Declined eGFR and albuminuria may not seldom predict the DN's extent when the serum creatinine levels have risen already. Therefore, a more effective indicator that can predict DN's progression is greatly warranted to deal with DN and, consequently, ESKD. In the present study, we found the HbA1c, GA, AGI index significantly associated with decreased kidney function. Both serum and urinary AGEs are significantly associated with adverse kidney function in these patients with DN. Elevated CML proportionate with reduced expression of podocyte-specific markers such as nephrin and podocin. Our study suggests that AGEs associate with EMT of podocytes and glomerulosclerosis in DN patients. Similarly, *in vivo* administration of AGEs resulted in podocyte EMT, glomerulosclerosis, and proteinuria. Inhibition of RAGE prevented AGEs induced adverse kidney effects both *in vivo* and *in vitro*, such as podocyte depletion, sclerosis, and proteinuria. Together, the data presented in our study demonstrate that AGEs may predict DN progression, particularly podocyte injury.

Chronic elevation of blood glucose levels is an exacerbating factor that ensures the non-enzymatic glycation and formation of AGEs, which deposit irreversibly in several organs and blood vessels (32). Serum levels of AGEs not only associate with the severity of diabetic complications, including retinopathy and nephropathy (33) but also predict mortality (34–37). In addition to predicting the risk of diabetic complications, Luft et al. reported that circulating CML levels predict the risk of developing diabetes (38). With each 100 ng/ml increment in CML the risk of developing diabetes increases by 35% in individuals with impaired fasting glucose (38). While in the American cohort circulating CML levels were associated with insulin resistance (HOMA-IR), in the Japanese cohort, no association was found for CML despite AGEs were associated with the HOMA-IR index (30, 39). AGEs elicit intracellular signaling events via interaction with RAGE localized to endothelial cells, macrophages, and vascular smooth muscle cells. The dominant AGE epitope for binding to the RAGE is CML (40). At the same time, CML modifications of proteins are predominant AGEs that accumulate *in vivo* (41). Elevated serum CML levels were observed in patients with kidney failure (42). Enhanced CML accumulation was observed in glomerular nodular lesions from patients with DN (43). AGEs-RAGE interaction elicits cellular injury by producing reactive oxygen species, activating profibrotic and proinflammatory cascades (12, 44). A recent report suggests that it may be necessary to evaluate glycemic control in patients with diabetes undergoing hemodialysis by combining several glycemic control indicators, including GA, HbA1c, and pre-dialysis blood glucose levels (45).

Infusion of AGEs into rats induced albuminuria, and histological changes like that occur during DN. Contrastingly, preventing AGEs formation improved proteinuria and preserved kidney function. DN is presented with reduced podocyte density. The specific effect of AGEs on podocyte biology is being investigated recently. AGEs, particularly CML, induce EMT of podocytes by inducing transcription factor Zeb2 through activation of NF-kB signaling cascade (2). At the same time, inhibition of NF-kB prevented CML-dependent induction of Zeb2 and loss of E-cadherin, which is crucial for maintaining epithelial morphology and cell-cell adhesion (2). A recent study showed that CML induced Notch signaling in podocytes contributing to their EMT (29). Administration of AGEs elicits decreased podocyte count in mice (29). AGEs accumulate in glomeruli and elicit the expression of ECM components such as type IV collagen and laminin. AGEs provoke premature senescence of the kidney cells, particularly cells in the proximal tubule. These novel actions of AGEs in eliciting podocytopathy vis-a-vis the pathogenesis of proteinuria and DN could be adapted as prognostic markers to assess the glomeruli's irreversible damage during the progression of DN.

HbA1c is the most used marker for glycemic control, and it is also used to predict the morbidity of vascular complications. HbA1c reflects plasma glucose levels for the past 2–3 months due to erythrocytes' long lifespan. However, certain clinical conditions such as kidney anemia and hemolytic anemia during which lifespan of erythrocytes vis-a-vis HbA1c measurements are affected and underestimate glycemic control. Furthermore, low hemoglobin levels may result in falsely low HbA1c values that underestimate glycemic control in patients undergoing dialysis. Increased hemoglobin turnover might contribute to lower glycated hemoglobin in advanced CKD and may mislead the clinical judgment. On the other hand, erythropoietin treatment in anemic patients with kidney disease significantly alters the HbA1c levels (23). Therefore, it is considered that over-reliance on HbA1c as the sole marker of glycemic control could lead to errors in assessing actual changes in glycemia (23). In this setting, an additional assessment of glycemic control is required.

AGI might represent a better glycemic control marker than HbA1c in diabetic patients with kidney insufficiency. In a recent study, Chitra et al. reported that plasma AGI was significantly (*P* < 0.05) associated with diabetic cataracts (46). In a recent study, Farhan et al. reported an association between AGEs/soluble RAGE ratio and urinary albumin/serum creatinine ratio in type II diabetic patients (47). Similar to our study, this study also suggests that serum AGEs can consider as predictors of vascular complications in uncontrolled type II diabetic patients. Therefore, markers that provide an index of long-term glycemic control are essential tools in DN patients' care, considering the increased incidence of DM and progression toward ESKD. In this study, we measured only one AGEs-CML. The association of other AGEs with podocyte injury may be similar as we observed or maybe different, which needs to be investigated. Another limitation a relatively small sample size. Our subjects were abnormally hyperglycemic, and the data with diabetic patients with a good glycemic index may be different. A prolonged follow-up study with more patient numbers would make the present observation stronger. However, given the supportive findings from animal studies and biopsy samples, AGI's potential and measurement of individual AGEs could give a better index of progression of DN to ESKD. We are currently pursuing a study with an extended patient number for a longer duration. In summary, our study demonstrated that in proteinuria patients, glomerular CML levels correlate significantly with EMT of podocytes and glomerular injury. Administration of AGEs to mice provoked EMT of podocytes proved the cause and effect relationship between AGEs and glomerular injury. The findings suggest that CML could provoke podocyte injury and the risk of DN progression to ESKD. Therefore, serum and urinary AGI and CML might be considered a potential surrogate prognostic marker for DN.

## Data Availability Statement

The original contributions presented in the study are included in the article/supplementary material, further inquiries can be directed to the corresponding authors.

## Ethics Statement

The studies involving human participants were reviewed and approved by Guntur Medical College, Guntur General Hospital. The patients/participants provided their written informed consent to participate in this study. The animal study was reviewed and approved by University of Hyderabad.

## Author Contributions

RN, VT, KP, and AP planned and designed the study. RN, VT, and KP collected patients' data. RN, RK, MM, and AS performed the experiments. RN and AP evaluated the data. RN, VT, and MM performed the statistical analysis. RN and AP wrote the manuscript. All authors contributed to the article and approved the submitted version.

## Conflict of Interest

The authors declare that the research was conducted in the absence of any commercial or financial relationships that could be construed as a potential conflict of interest.
